# Association between Herpesviruses and Chronic Periodontitis: A Meta-Analysis Based on Case-Control Studies

**DOI:** 10.1371/journal.pone.0144319

**Published:** 2015-12-14

**Authors:** Ce Zhu, Fei Li, May. Chun. Mei Wong, Xi-Ping Feng, Hai-Xia Lu, Wei Xu

**Affiliations:** 1 Department of Preventive Dentistry, Ninth People’s Hospital, School of Medicine, Shanghai Jiao Tong University, Shanghai Key Laboratory of Stomatology, Shanghai, China; 2 Dental Public Health, Faculty of Dentistry, University of Hong Kong, Hong Kong, China; 3 Department of Preventive Dentistry, Shanghai Municipal Hospital for Oral Health, Shanghai, China; University of Nebraska - Lincoln, UNITED STATES

## Abstract

**Objective:**

Numerous studies have investigated the associations between herpesviruses and chronic periodontitis; however, the results remain controversial. To derive a more precise estimation, a meta-analysis on all available studies was performed to identify the association between herpesviruses and chronic periodontitis.

**Methods:**

A computerized literature search was conducted in December 2014 to identify eligible case-control studies from the PUBMED and EMBASE databases according to inclusion and exclusion criteria. Data were extracted and pooled odds ratios (OR) with 95% confidence intervals (CI) were used to assess the association between herpesviruses and risk of chronic periodontitis. A fixed or random effects model was determined based on a heterogeneity test. Sensitivity analysis was conducted to investigate stability and reliability. Publication bias was investigated using the Begg rank correlation test and Egger's funnel plot.

**Results:**

Ten eligible studies were included to investigate the association between Epstein–Barr virus (EBV) and chronic periodontitis. The results showed that EBV has a significant association with chronic periodontitis compared with periodontally healthy group (OR = 5.74, 95% CI = 2.53–13.00, *P*<0.001). The association between human cytomegalovirus (HCMV) and chronic periodontitis was analyzed in 10 studies. The pooled result showed that HCMV also has a significant association with chronic periodontitis (OR = 3.59, 95% CI = 1.41–9.16, *P* = 0.007). Similar results were found in the sensitivity analyses. No significant publication bias was observed. Two eligible studies were included to investigate the association between herpes simplex virus (HSV) and chronic periodontitis risk. The association between HSV and chronic periodontitis was inconclusive (OR = 2.81 95% CI = 0.95–8.27, *P* = 0.06). Only one included study investigated the association between human herpesvirus 7 (HHV-7) and chronic periodontitis risk (OR = 1.00, 95% CI = 0.21–4.86).

**Conclusion:**

The findings of this meta-analysis suggest that two members of the herpesvirus family, EBV and HCMV, are significantly associated with chronic periodontitis. There is insufficient evidence to support associations between HSV, HHV-7 and chronic periodontitis.

## Introduction

Periodontitis is a complex disease that is among the most prevalent microbial diseases and chronic inflammatory diseases worldwide [1]. Chronic periodontitis is the most prevalent subgroup of periodontitis and can result in periodontal destruction, alveolar bone resorption, occasional pain, and eventual tooth loss [1]. Although the process of periodontitis is considered to involve a multifactorial interaction between microbial, host, and environmental modulating factors [2], microbial agents are of key importance in the development of periodontitis.

It is well accepted that periodontitis is associated with colonization of specific bacterial species upon the teeth surfaces. However, several studies have reported the absence of these specific bacterial species in patients with periodontal disease, and no significant difference has been found in the prevalence of bacteria between healthy and diseased periodontal tissues [3–4]. It is becoming increasingly clear that some major clinical characteristics of periodontitis, such as, site specificity, self-limited progression and recurrence, are difficult to be explained simply by the theory of bacterial infection [5]. In view of this issue and the fact that most evidence for the bacterial etiology of periodontitis is indirect, some scholars have suggested that the pure bacterial cause for periodontitis had been overemphasized [6].

Herpesviruses came into our sight as putative pathogens of periodontal disease in the mid 1990s. Herpesviruses are the most common viruses in humans, infecting 80–90% of the global adult population [7]. Eight members of the herpes family are known to cause human disease. These are Epstein-Barr virus (EBV), human cytomegalovirus (HCMV), herpes simplex virus 1 and 2 (HSV-1, HSV-2), varicella zoster virus (VZV), human herpesvirus 6 (HHV-6), human herpesvirus 7 (HHV-7), and human herpesvirus 8 (HHV-8). In the past decade, a number of studies have been conducted to investigate the influence of herpesvirus on the clinical characteristics of chronic periodontitis [8–19]. These findings were controversial regarding detection of the viral DNA of herpesviruses in the periodontal environment. Some studies indicated a significantly increased risk of chronic periodontitis accompanied by higher prevalence of detection of herpesvirus [9,10,12,13,16,19], whereas other studies indicated contrary results [8,11,14,15,17,18]. Hence, the goal of this study was to clarify the association between detection of herpesviruses and risk of chronic periodontitis through conducting a meta-analysis based on relevant case-control studies.

## Materials and Methods

This study was in compliance with the Preferred Reporting Items for Systematic Reviews and Meta-Analyses (PRISMA) statement guidelines. A review protocol did not exist.

### Search strategy

A computerized literature search of the PUBMED and EMBASE databases to identify relevant case-control studies that assessed the relationships between herpesviruses and chronic periodontitis was conducted in December 2014 by two reviewers (Ce Zhu and Fei Li). The following phrases or keywords were used: (virus OR herpesvir* OR EBV OR CMV OR HCMV OR HHV OR HSV OR Epstein-Barr virus OR cytomegalovirus OR herpes simplex virus OR Human herpesvirus) AND (chronic periodontitis OR periodontal disease OR paradontosis OR parodontopathy OR periodontal). The search was performed in English without any other restrictions. A manual search involved the reference lists of the relevant articles retrieved was also conducted.

### Inclusion and exclusion criteria

The included studies met the following inclusion criteria: (1) studies designed as a case-control study (patients with chronic periodontitis versus periodontally healthy volunteers); (2) studies evaluating the association between herpesviruses and chronic periodontitis; (3) sample locations were: subgingival plaque, gingival crevicular fluid, and biopsy;(4) methods of sample analyses were: polymerase chain reaction (PCR), real-time PCR, nested PCR, or multiplex PCR; (5) all participants were systemically healthy; (6) sufficient data could be extracted from articles, obtained by calculation, or obtained by contacting the authors of the primary studies.

Studies meeting more than one of the following criteria were excluded: (1) case reports, review articles, animal studies, or studies that were not case-control design; (2) samples were obtained from participants with systemic disease; (3) saliva samples were used for detection of the selected viruses; (4) available data could not be extracted or obtained by calculation from the article or by contacting the authors of the primary studies; (5) duplicate publication.

### Study selection

The title and abstract of retrieved articles were screened to ascertain potential inclusion. The full article was obtained for articles appearing to qualify for possible inclusion. After the assessment of full-text, those articles meeting all inclusion and exclusion criteria were submitted for final inclusion by reviewers.

### Data extraction

Data extraction from included studies was conducted in duplicate by the same two reviewers. The following information was extracted: first author’s name, publication year, country of the study population, demographics, total number of cases and controls, sample type, methods of sampling and sample analyses, and detection rate of herpesviruses. The detection rate of herpesviruses in case and control groups were extracted from the articles or obtained by calculation. Authors were contacted directly if crucial data were not reported in the original articles. Any controversial issues were resolved by discussion.

### Statistical analysis

The statistical analysis was performed using RevMan version 5.1 (The Nordic Cochrane Centre, The Cochrane Collaboration, Copenhagen, Denmark) and Stata Release 12 (StataCorp, College Station, TX, USA) software. Odds ratios (OR) and the corresponding 95% confidence intervals (CI) were used to measure the association between detection of herpesviruses and chronic periodontitis risk. Heterogeneity test was assessed with use of the Q test and I^2^ value. A fixed or random effects model was selected based on a heterogeneity test: if the *P* value of Q statistic was less than 0.1 or I^2^ was more than 50%, the random effects model was chosen; otherwise, the fixed effects model was chosen. Additionally, a sensitivity analysis was conducted to access the effect of a single study on the pooled ORs by omitting one study in turn each time. The publication bias was investigated with the Begg rank correlation test and Egger's funnel plot, the Egger's funnel plot asymmetry was assessed using regression liner method. *P*<0.05 was considered statistically significant and all *P*-values were two-sided.

## Results

### Description of the studies

**Fig 1:**
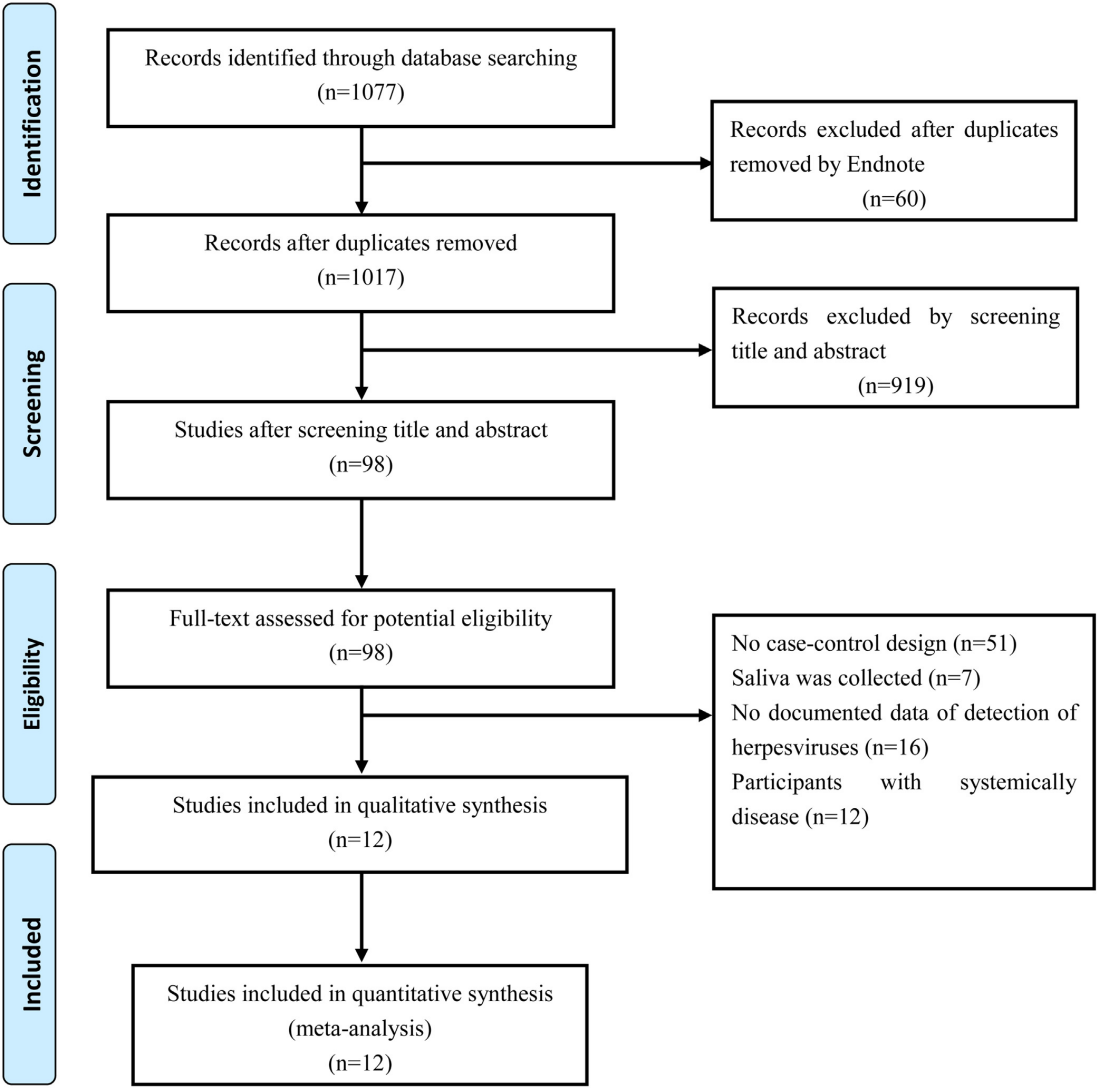
Flow chart for the study selection process.

We initially identified 1077 records from the above databases, with 1017 records remaining after duplicates were removed in EndNote. A total of 919 non-relevant records were excluded by screening the titles and abstracts. The full-texts of the remaining 98 studies were treated in detail, of which 12 relevant studies involving the association between herpesviruses and chronic periodontitis were eligible according to the above inclusion and exclusion criteria. Eventually, a total of 12 studies comprising 552 cases and 371 controls were included in this meta-analysis. The study selection process is shown in Fig 1, and the characteristics are summarized in Table 1.

**Table 1:** Characteristics of studies included in the meta-analysis.

Studies	Country	Participants(cases/controls)		Materials and Methods			Herpesviruses Prevalence	
		Sample Size	Mean Age	Sample Type	Sampling	PCR type	Cases	Controls
Saygun et al, 2002 [8]	Turkey	30/21	42.80/41.72	SP	Paper point	Nested PCR	EBV(+)5/30 HCMV(+)13/30 HSV(+) 2/30	EBV(+)3/21 HCMV(+)3/21 HSV(+) 0/21
Wu et al, 2006 [9]	China	65/24	Males 43.9/37.3 Femals45.3/36.3	SP	Paper point	Nested PCR	EBV(+) 43/65	EBV(+) 4/24
Botero et al, 2007 [10]	Colombia	20/22	44.0/31.2	SP	Paper point	Nested PCR	HCMV(+) 12/20	HCMV(+) 4/22
Wu et al, 2007 [11]	China	143/76	Males 41.4/38.8 Femals42.5/37.5	SP	Paper point	Nested PCR	EBV(+)91/143 HCMV(+)113/143	EBV(+)23/76 HCMV(+)58/76
Chalabi et al, 2008 [12]	Iran	61/40	42.9/40.7	SP	Curette	Nested PCR	EBV(+)48/61 HCMV(+) 35/61	EBV(+)1/40 HCMV(+) 1/40
Imbronito et al, 2008 [13]	Brazil	30/30	42.7/28.1	SP	Paper point	Nested PCR	EBV(+)14/30 HCMV(+)15/30 HSV(+) 12/30	EBV(+)0/30 HCMV(+)17/30 HSV(+) 6/30
Rotola et al, 2008 [14]	Italy	13/13	50.8/25.8	BS	Biopsy	Nested PCR	EBV(+)6/13 HCMV(+)0/13 HHV-7(+) 8/13	EBV(+)1/13 HCMV(+)1/13 HHV-7(+) 8/13
Nibali et al, 2009 [15]	UK	20/40	43.4/50.3	SP	Curette	Real-time PCR	EBV(+)0/20 HCMV(+) 0/20	EBV(+)4/40 HCMV(+) 0/40
Chalabi et al, 2010 [16]	Iran	40/40	40.9/42.0	SP	Curette	PCR	HCMV(+) 20/40	HCMV(+) 2/40
Das et al, 2012 [17]	India	25/25	N/A	SP	Curette	Multiple PCR	EBV(+)8/25 HCMV(+) 7/25	EBV(+)2/25 HCMV(+) 2/25
Sharma et al, 2012 [18]	India	20/20	42.53/36.52	SP	Curette	PCR	EBV(+)5/20 HCMV(+) 4/20	EBV(+)0/20 HCMV(+) 2/20
Kato et al, 2013 [19]	Japan	85/20	57.4/45.9	SP	Paper point	Nested PCR	EBV(+) 56/85	EBV(+) 18/40

Abbreviations: N/A, not applicable; SP, subgingival plaque; BS, biopsy specimen; PCR, polymerase chain reaction.

### Quantitative synthesis

**Fig 2:**
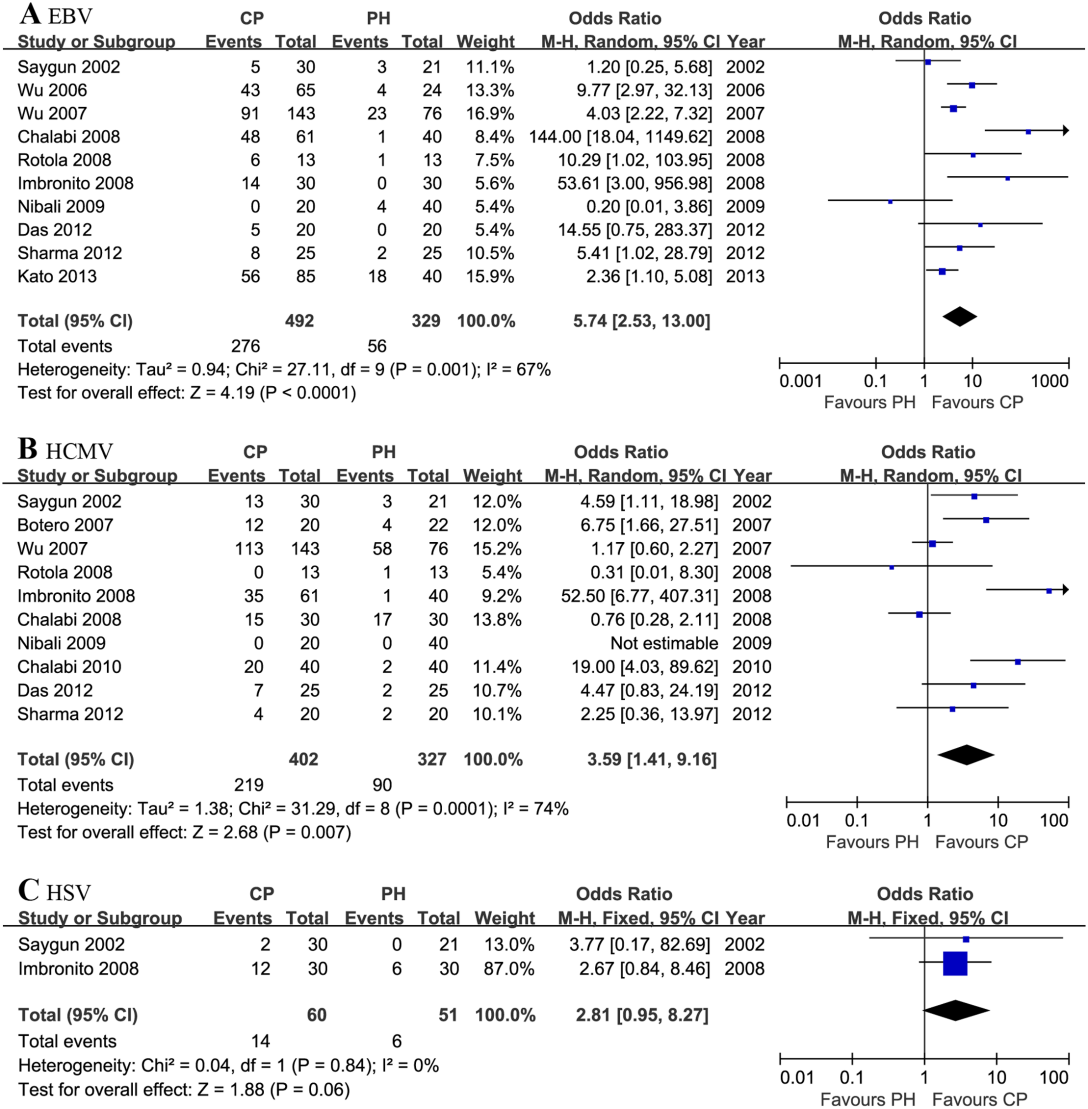
Forest plot of the association between herpesviruses and risk of chronic periodontitis (CP): (A) EBV and CP risk, (B) HCMV and CP risk, (C) HSV and CP risk.

Quantitative synthesis was treated separately according to herpesvirus types. Forest plots of the association between herpesviruses and risk of chronic periodontitis are shown in Fig 2. The association between EBV and chronic periodontitis was analyzed in 10 studies involving 492 patients with chronic periodontitis and 329 periodontally healthy controls. The heterogeneity was significant (*P*_*h*_<0.001, I^2^ = 67%), so the random effects model was selected. The pooled results showed that EBV has an association with a significantly increased risk of chronic periodontitis (OR = 5.74, 95% CI = 2.53–13.00, *P*<0.001) (Fig 2A).

The association between HCMV and risk of chronic periodontitis was analyzed in 10 studies involving 402 patients with chronic periodontitis and 327 periodontally healthy controls. The random effects model was also selected according to the heterogeneity test (*P*_*h*_<0.001, I^2^ = 74%). The pooled results showed that HCMV has an association with a significantly increased risk of chronic periodontitis (OR = 3.59, 95% CI = 1.41–9.16, *P* = 0.007) (Fig 2B).

Two eligible studies were included to investigate the association between HSV and chronic periodontitis risk. The fixed effects model was selected according to the heterogeneity test (*P*_*h*_ = 0.84, I^2^ = 74%). The association between HSV and chronic periodontitis was inconclusive (OR = 2.81 95% CI = 0.95–8.27, *P* = 0.06) (Fig 2C).

Only one included study investigated the association between HHV-7 and chronic periodontitis risk (Rotala et al. 2008; OR = 1.00, 95% CI = 0.21–4.86). No included study reported detection of VZV, HHV-6 and HHV-8 in the selected samples.

### Sensitivity analysis

**Fig 3:**
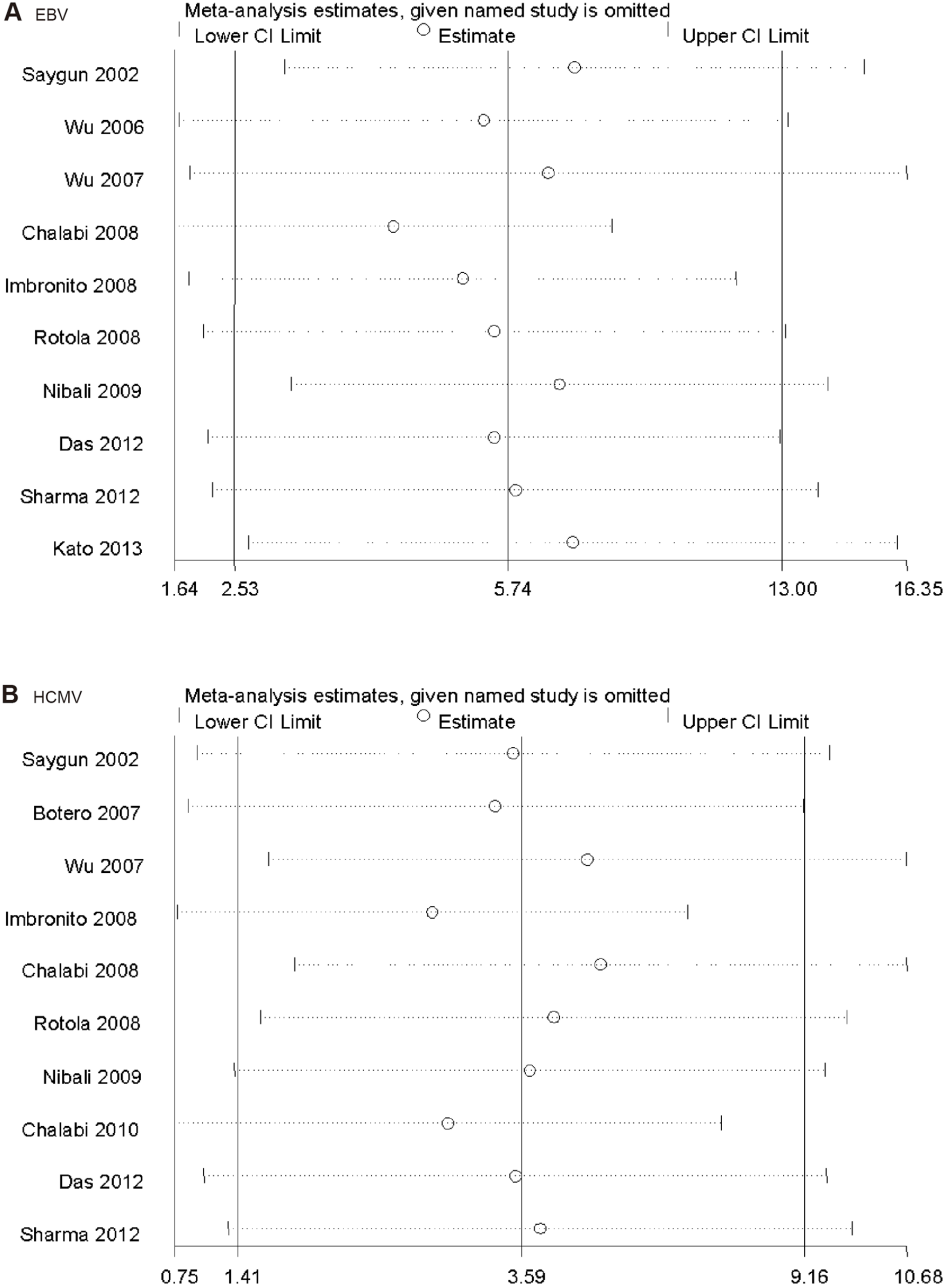
Sensitivity analysis of the association between herpesviruses and risk of chronic periodontitis (CP): (A) EBV and CP risk, (B) HCMV and CP risk.

Results of sensitivity analysis of the associations between EBV, HCMV, and chronic periodontitis are presented in Fig 3. The pooled results yielded consistent results when any single study was omitted. In the overall meta-analysis, no single study changed the pooled ORs significantly, suggesting that the results were statistically stable and reliable. Because of the limited number of articles, we did not assess the sensitivity analysis for the other members of the herpesvirus family.

### Publication bias

**Fig 4:**
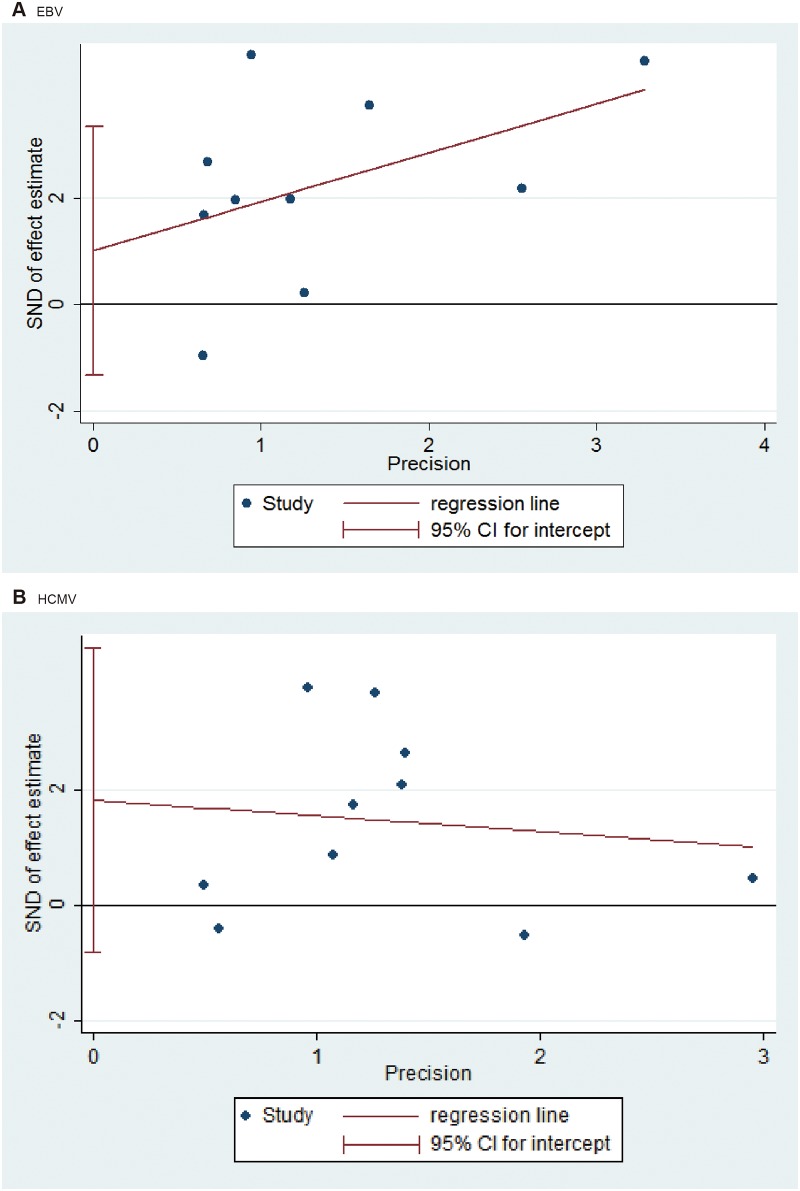
Egger’s funnel plot of the association between herpesviruses and risk of chronic periodontitis (CP): (A) EBV and CP risk, (B) HCMV and CP risk.

As shown in Fig 4, no significant publication bias was indicated in this meta-analysis by both the Begg rank correlation test and the Egger’s funnel plot asymmetry test (regression liner method) (EBV: Begg test, *P* = 0.754; Egger test, *P* = 0.345. HCMV: Begg test, *P* = 0.721; Egger test, *P* = 0.151). Because of the limited number of articles, we did not assess the publication bias for the other members of the herpesvirus family.

## Discussion

Periodontitis affects the majority of adults worldwide [20]. The main etiological agents are varied microbial species organized in form of biofilm. The influence of periodontopathic bacteria on the pathogenesis of periodontitis has been well recognized [21]. Although the presence of specific Gram-negative bacteria is essential to initiatie and perpetuate periodontal disease, a bacterial etiology alone cannot explain the clinico-pathologic features presented in the disease [22]. Evidence convincingly suggests that the presence of certain viruses in the periodontal environment promote the course of periodontal disease, and possible mechanisms have also been suggested. Millions of genomic copies of human viruses may be harbored in periodontal lesions [7], with herpesviruses the most commonly researched viruses in periodontology [23]. Slots [23] presented evidence that viruses and bacteria in aggregate can produce a greater pathogenic effect than the sum of the individual agents, and the current model of the pathogenesis of periodontitis should be revisited according to the concept of a herpesviral-bacterial co-infection.

A number of studies have reported a significant relationship between herpesviruses and risk of chronic periodontitis; however, the findings remain inconsistent. Several groups have provided weak associations and even opposite results between periodontal herpesviruses and chronic periodontitis [8,11,12,14,15]. A recent review has listed the findings on herpesviruses detected in patients with periodontal disease and periodontally healthy people, and summarized pathogenic characteristics of the herpesviral-bacterial co-infection model [24]. Another review described in more detail on the interaction between herpesviruses and putative periodontal bacteria [25]. However, no meta-analysis has given overall estimated results on the association between herpesviruses and risk of chronic periodontitis. Hence, we performed the first meta-analysis to clarify this research question based on existing research data. These findings may help to provide more reliable evidence than single case-control studies.

The combined results of this meta-analysis reveal evidence that EBV and HCMV have a significant association with increased risk of chronic periodontitis. The sensitivity analyses indicated that the pooled estimates were comparatively robust, and no significant publication bias was observed. There is insufficient evidence to support the associations between HSV, HHV-7 and chronic periodontitis because only two eligible studies for HSV and one eligible study for HHV-7 were included in this review. Further high quality studies based on large scale samples are needed to identify the association with chronic periodontitis for HSV and HHV-7. There were no included studies covering the remaining members of the herpes virus family.

The majority of studies compared the viral detection frequency among chronic periodontitis patients and periodontally healthy volunteers. Relationships were investigated between the detection of herpesviruses and clinic parameters, including probing depth and attachment loss. Most studies detected HCMV genomes in more than 50% of infected sites [10–13,16]; the sampling sites of control groups generally showed lower frequency of viral detection [8,10,12,14–18]. However, two studies reported higher occurrence of HCMV in the control group [11,13], and three studies only detected HCMV in less than 25% of infected sites [14,15,18]. Of 10 studies addressing the presence of EBV, half reported a detection frequency of more than 50% in the infected sites of patients with chronic periodontitis [9,11,12,14,19], another five studies failed to detect such high EBV occurrence among such patients [13,15,17,18,26]. All studies on periodontally healthy volunteers reported occurrence of EBV in more than 50% of participants.

Periodontitis involves inflammatory processes characterized by accumulation of immune cells [27]. Previous research has suggested that because of viral replication in lymphocytes, monocytes and macrophages, viruses can adjust to immune mechanisms and influence the immune response directly or indirectly [28,29]. Conceivably, herpesviruses can reduce the ability of periodontal tissues to resist bacterial invasion through infecting or alterring structural cells or host defense cells of the periodontium [30–32]. Periodontitis may be evoked by herpesviral-bacterial co-infection.

The mechanisms of EBV and HCMV in the pathogenesis of periodontitis remain unclear. EBV primarily infects B-lymphocytes, and HCMV infects several types of cells. HCMV could establish latency in macrophage granulocyte progenitor cells and peripheral blood mononuclear cells [33]. Evidence supported that EBV and HCMV are related to the subgingival presence of major periodontal pathogenic bacteria. Saygun et al. [26] revealed a close relationship between counts of *Porphyromonas gingivalis* and *Tannerella forsythia* and genome copy-counts of EBV and HCMV. Another study revealed that EBV reactivation could be induced by P. gingivalis through epigenetic regulation [31]. EBV-HCMV co-infection tends to be associated with severe clinical features in chronic periodontitis [11,12].

Data evaluating the association between detection of herpesviruses and risks of other periodontal disease entities weren’t included in this study. The aim of this meta-analysis was to statistically analyze the association between detection of herpesviruses and risk of chronic periodontitis; therefore, we included 12 studies investigating this association. Unfortunately, among those studies, only three detected the herperviral-bacterial co-infection in chronic periodontitis. Betero et al. [10] found that *Prevotella intermedia*, *Prevotella nigrescens*, *and Eikenella corrodens* were increased in HCMV-positive patients with chronic periodontitis (*P <* 0.05), suggesting that simultaneous infection with high levels of periodontopathic microorganisms and HCMV could increase the risk for periodontitis. Imbronito et al. [13] found that coinfections of EBV-1 and *P*. *gingivalis*, HCMV and *T*. *forsythia*, HCMV and *Aggregatibacter actinomycetemcomitans*, HSV-1 and *T*. *forsythia*, as well as HSV-1 and *P*. *intermedia* were more frequent in patients with chronic periodontitis than in periodontally healthy individuals (*P*<0.05). Kato et al. [19] found that the ORs of having chronic periodontitis (PD≥5 mm) for EBV and *P*. *gingivalis* coinfection were higher (approximately 1.82-fold) compared with the solitary presence of either EBV DNA or *P*. *gingivalis*. All groups of researchers suggested herpesviral-bacterial co-infection may increase the risk for periodontitis, but definite conclusions could not be drawn owing to insufficient sample size. Chalabi et al. [16] detected both periodontopathic bacteria and herpesviruses in chronic periodontitis sites, but analysis for coinfections was not performed. The remaining eight included studies did not investigate periodontopathic bacteria in the selected samples. Especially, the mechanisms of herpesviral-bacterial co-infection are not entirely clear. In view of these facts, this meta-analysis estimated the OR of herpesviruses infection only. Quantitative synthesis based on data evaluating the association between herpesviral-bacterial co-infection and risk of periodontal disease is required. This meta-analysis discussed herpesviruses and chronic periodontitis risk in systemically healthy people. Syndromes present with advanced types of periodontitis may serve as a model for studying herpesviruses in the pathogenesis of periodontal disease [34–36].

Several limitations should be considered in this meta-analysis. First, significant heterogeneity between the studies included in quantitative synthesis for EBV and HCMV were detected. However, we were unable to conduct subgroup analysis based on sample type, Sampling method, or analysis method owing limited data. Second, a language bias might be a potential problem because the included studies were all published in English. Third, only two studies were included in the quantitative synthesis for HSV, which may have provided insufficient power to test for a significant association. Fourth, the age distributions of participants were extracted from each included study, but the confounding effect of age was not evaluated in this meta-analysis. Last, the results of our meta-analysis were applicable to only three ethnic groups—Europeans, Asians, and ethnic groups of the Americas; there were no relevant studies conducted among African ethnic groups.

Pathogenesis research has established system theories about the promotion effect of herpesviruses on periodontitis. Results of the present meta-analysis also suggest that EBV and HCMV have an association with significantly increased risk for chronic periodontitis. However, current periodontal therapy, which has been proved to be ineffective in treating late stage disease, is still based on the concept of traditional bacterial infection. The lack of a curative effect might be owing to the failure to apply viral pathogenesis theory [37]. Sunde et al. [38] treated a patient with refractory periodontitis and high EBV levels using valacyclovir hydrochloride, resulting in marked improvement of the patient’s periodontal situation. Cobb et al. [6] also found that the application of antiviral interferon could reduce gingival inflammation in a canine model. Periodontal treatment that includes both antibacterial debridement and antiviral agents might be more effective in treating the intractable and recurrent periodontitis. Further studies may provide more data on EBV and HCMV as factors in the pathogenesis of chronic periodontitis, and new approaches to preventing and treating chronic periodontitis may focus on controlling herpesviruses. This meta-analysis provided circumstantial evidence that herpesviruses play a role in chronic periodontitis; nevertheless, a cause-and-effect relationship remains to be established. The possible involvement of human herpesviruses in the pathogenesis of chronic periodontitis merits further investigation.

## Supporting Information

S1 PRISMA Checklist PRISMA Checklist. (DOC)
